# Supplementary material for: Transcriptomic analyses of the termite, Cryptotermes secundus, reveal a gene network underlying a long lifespan and high fecundity
Source: Commun Biol. 2021 Mar 22;4:384. doi: 10.1038/s42003-021-01892-x (PMC7985136; doi:10.1038/s42003-021-01892-x)
Supplement: Supplementary file 2 — Supplementary Information [file 42003_2021_1892_MOESM2_ESM.pdf]

Supplementary Information for:

Title: Transcriptomic analyses of the termite, *Cryptotermes secundus*, reveal a gene network underlying a long lifespan and high fecundity

Authors: Silu Lin[1,+], Jana Werle[1,+], Judith Korb[1,*]
[+]these authors contributed equally

Affiliations:
[1] Evolutionary Biology & Ecology, University of Freiburg, Freiburg, Germany

***Corresponding Author:**
judith.korb@biologie.uni-freiburg.de

This PDF file includes:

Supplementary Methods
Supplementary Figures 1
Supplementary Figure 2 a, b
Supplementary References

Other supplementary materials for this manuscript include the following:

Supplementary Data 1 to 7

Supplementary Methods

Processing of RNASeq raw reads. We trimmed adapters and kept sequences with a minimum length of 70 bases. For the queen network experiment, raw reads were trimmed using fastp[1] (version 0.19.6) with the parameters -l 70, --thread 5, -Q and others as default. For the JH manipulation experiment, raw reads were trimmed using Trimmomatic[2] (version 0.38) in paired-end mode using the ILLUMINACLIP default setting.

Supplementary Figures

Supplementary Figure 1

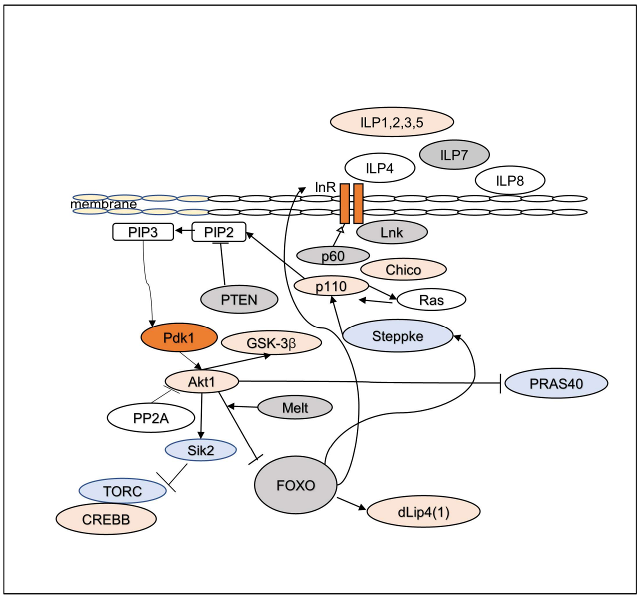

Supplementary Figure 1. Schematic representation of the IIS (insulin/insulin-like-growth factor 1 signaling) pathway compiled from studies on *Drosophila*[3–5]. Shown is how different genes associate with *Cryptotermes secundus* queens as revealed from WGCNA. The data summarizes the results of experiment 1 (i.e., the comparison of gene expression profiles between queens and workers) for IIS pathway genes for all detected modules (see Supplementary Data 3a), not only the QCM. Arrows indicate direct activation, stop bars indicate inhibition. Associations of genes with the queen phenotype are indicated by colors. Orange indicates a positive association with queens (light orange: not significant; bright orange: significant). Blue indicates negative associations with queens (i.e., typical for workers), yet none of them was significant. Grey indicates genes that were not co-expressed; therefore, gene-queen associations were not calculated. PP2A and Ras both are multi copy genes in *C. secundus* with different copies showing different directions. Therefore, a color cannot be assigned. There are only two ILPs in cockroaches, incl. termites. One (*Csec-ILP7*) seems to be an ortholog of dilp7, and one, Csec_G03496, is similar to dilp1,2,3,5, from *D. melanogaster*. *PIP2*, *PIP3*, *ILP4* and *ILP8* genes are unknown in *C. secundus*; therefore, no color can be assigned. There are three described *InR* genes in *C. secundus* (*Csec-InR-1*, *Csec-InR-2*, *Csec-InR-3*)[6] and an additional one, *Csec_G15826*, identified in this study. All *InR* genes were not members of the QCM except for *Csec_G15826*. However, they were all associated with queens. *Csec_G15826* was significantly associated with queens and *Csec-InR-2* by trend (see also Figure 3). *Csec-PDK1* and the *InR* gene *Csec_G15826* were also among the DEGs, significantly upregulated in queens (see Supplementary Data 2a).

Supplementary Figure 2

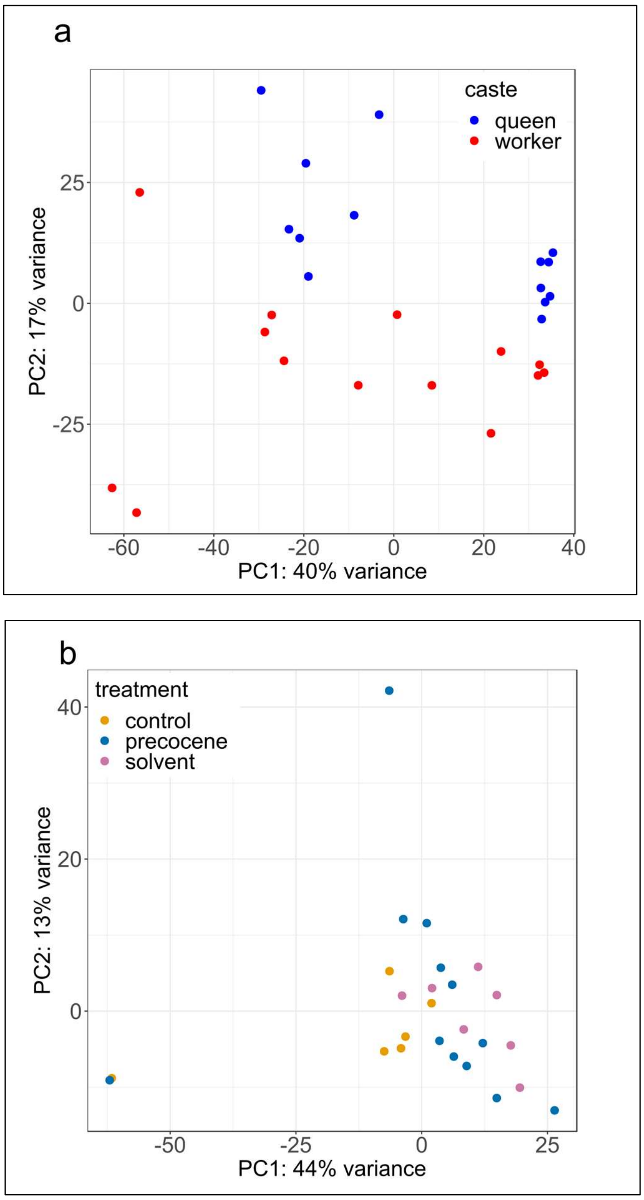

Supplementary Figure 2. Results of the Principal Component Analysis of the gene expression studies. Each dot represents the principal component (PC) scores of a sample. Color represents the 'character' (caste, treatment) of the sample. (a) PCA plot for 14 queens and 14 workers of the queen network experiment. Castes were separated by PC2, which explained 17% of the variance. (b) PCA plot for 12 precocene-treated, 7 solvent-treated and 6 untreated control queens in the JH manipulation experiment.

Supplementary References

1. Chen, S., Zhou, Y., Chen, Y. & Gu, J. Fastp: An ultra-fast all-in-one FASTQ preprocessor. in *Bioinformatics* vol. 34 i884–i890 (Oxford University Press, 2018).
2. Bolger, A. M., Lohse, M. & Usadel, B. Trimmomatic: a flexible trimmer for Illumina sequence data. *Bioinformatics* **30**, 2114–2120 (2014).
3. Partridge, L., Alic, N., Bjedov, I. & Piper, M. D. W. Ageing in Drosophila: The role of the insulin/Igf and TOR signalling network. *Experimental Gerontology* **46**, 376–381 (2011).
4. Kučerová, L. *et al.* Slowed aging during reproductive dormancy is reflected in genome-wide transcriptome changes in Drosophila melanogaster. *BMC Genomics* **17**, (2016).
5. Stanley, P. D., Ng'oma, E., O'Day, S. & King, E. G. Genetic dissection of nutrition-induced plasticity in insulin/insulin-like growth factor signaling and median life span in a Drosophila multiparent population. *Genetics* **206**, 587–602 (2017).
6. Kremer, L. P. M., Korb, J. & Bornberg-Bauer, E. Reconstructed evolution of insulin receptors in insects reveals duplications in early insects and cockroaches. *Journal of Experimental Zoology Part B: Molecular and Developmental Evolution* **330**, 305–311 (2018).
