# Supplementary material for: Transcriptomic analyses of the termite, Cryptotermes secundus, reveal a gene network underlying a long lifespan and high fecundity
Source: Commun Biol. 2021 Mar 22;4:384. doi: 10.1038/s42003-021-01892-x (PMC7985136; doi:10.1038/s42003-021-01892-x)
Supplement: Supplementary file 3 — Description of Supplementary Files [file 42003_2021_1892_MOESM3_ESM.pdf]

Description of Additional Supplementary Files

File name: Supplementary Data **1**

Description: Detailed overview of Cryptotermes secundus (Isoptera, Kalotermitidae) samples used for RNAseq analyses, sorted by experiment.

File name: Supplementary Data 2

Description: Differentially expressed genes (DEGs) (after FDR correction) (A) between queens and workers of the queen network experiment; (B) between control & precocene-treated queens of the JH manipulation experiment; (C) between control and acetone-treated queens of the JH manipulation experiment; (D) between acetone & precocene-treated queens of the JH manipulation experiment.

File name: Supplementary Data 3

Description: Modules of the WGCNA. (A) 176 modules of the queen network experiment aimed to characterize queens versus workers; (B) 224 modules of the precocene-effect analysis: precocene-treated queens versus control queens; (C) 219 modules of the solvent effect analysis: solvent treated queens versus control queens; (D) 216 modules of the analysis aimed to disentangle the JH effect: Precocene versus solvent treated queens.

File name: Supplementary Data 4

Description: List of the 99 TI-J-LiFe genes (for TOR/IIS-JH-Lifespan and Fecundity). This is a list of genes from the TI-J-LiFe network that combines all major pathways underlying aging and life history trade-offs. This list was originally generated for D. melanogaster (see Main text).

File name: Supplementary Data 5

Description: KEGG enrichment results for all modules from different WGCNA comparisons. (A) Queen network experiment: queens versus workers; (B) Precocene effect: precocene-treated queens versus control queens; (C) Solvent effect: solvent treated queens versus control queens; (D) JH effect: Precocene versus solvent treated queens.

File name: Supplementary Data 6

Description: Gene ontology (GO) enrichment results for all modules from different WGCNA. (A) Queen network experiment: queens versus workers; (B) Precocene effect: precocene-treated queens versus control queens; (C) Solvent effect: solvent treated queens versus control queens; (D) JH effect: Precocene versus solvent treated queens.

File name: Supplementary Data 7

Description: Results of Blast searches against the cockroach Blattella germanica and the termite Zootermopsis nevadensis.
