# Supplementary material for: Transcriptomic analyses of the termite, Cryptotermes secundus, reveal a gene network underlying a long lifespan and high fecundity
Source: Commun Biol. 2021 Mar 22;4:384. doi: 10.1038/s42003-021-01892-x (PMC7985136; doi:10.1038/s42003-021-01892-x)
Supplement: Supplementary file 11 — Reporting Summary [file 42003_2021_1892_MOESM11_ESM.pdf]

nature research

Corresponding author(s): Judith Korb

Last updated by author(s): Jan 29, 2021

Reporting Summary

Nature Research wishes to improve the reproducibility of the work that we publish. This form provides structure for consistency and transparency in reporting. For further information on Nature Research policies, see our Editorial Policies and the Editorial Policy Checklist.

Statistics

For all statistical analyses, confirm that the following items are present in the figure legend, table legend, main text, or Methods section.

n/a	Confirmed	
☐	☒	The exact sample size (*n*) for each experimental group/condition, given as a discrete number and unit of measurement
☐	☒	A statement on whether measurements were taken from distinct samples or whether the same sample was measured repeatedly
☐	☒	The statistical test(s) used AND whether they are one- or two-sided<br>*Only common tests should be described solely by name; describe more complex techniques in the Methods section.*
☐	☒	A description of all covariates tested
☐	☒	A description of any assumptions or corrections, such as tests of normality and adjustment for multiple comparisons
☐	☒	A full description of the statistical parameters including central tendency (e.g. means) or other basic estimates (e.g. regression coefficient) AND variation (e.g. standard deviation) or associated estimates of uncertainty (e.g. confidence intervals)
☐	☒	For null hypothesis testing, the test statistic (e.g. *F*, *t*, *r*) with confidence intervals, effect sizes, degrees of freedom and *P* value noted<br>*Give P values as exact values whenever suitable.*
☒	☐	For Bayesian analysis, information on the choice of priors and Markov chain Monte Carlo settings
☒	☐	For hierarchical and complex designs, identification of the appropriate level for tests and full reporting of outcomes
☒	☐	Estimates of effect sizes (e.g. Cohen's *d*, Pearson's *r*), indicating how they were calculated

Our web collection on statistics for biologists contains articles on many of the points above.

Software and code

Policy information about availability of computer code

Data collection	No software was used.
Data analysis	We used: R (v.3.5.2), fastp (v.0.19.6), Trimmomatic (v.0.38), MultiQC (v.1.7), FastQC (v.0.11.5), HISAT2 (v.2.1.0), SAMtools (v.1.9), HTSeq (v.2015), DESeq2 (v.1.18.1), flashClust (v.1.01-2), WGCNA (v.1.68), dynamicTreeCut (v.1.63.1), InterProScan (v.5.33-72.0), BLASTP (v.2.4.0), TopGO (v.2.32.0), clusterProfiler (v.3.8.1)

For manuscripts utilizing custom algorithms or software that are central to the research but not yet described in published literature, software must be made available to editors and reviewers. We strongly encourage code deposition in a community repository (e.g. GitHub). See the Nature Research guidelines for submitting code & software for further information.

Data

Policy information about availability of data

All manuscripts must include a data availability statement. This statement should provide the following information, where applicable:
- Accession codes, unique identifiers, or web links for publicly available datasets
- A list of figures that have associated raw data
- A description of any restrictions on data availability

The raw sequence data used in this publication are deposited at the National Center for Biotechnology Information (NCBI) under Umbrella Project "SoLong", Bioproject ID PRJNA654896, PRJNA683654, PRJNA685294.

Field-specific reporting

Please select the one below that is the best fit for your research. If you are not sure, read the appropriate sections before making your selection.

☒ Life sciences ☐ Behavioural & social sciences ☐ Ecological, evolutionary & environmental sciences

For a reference copy of the document with all sections, see nature.com/documents/nr-reporting-summary-flat.pdf

Life sciences study design

All studies must disclose on these points even when the disclosure is negative.

Sample size	Sample size was chosen upon availability of termite individuals.
Data exclusions	No data were excluded.
Replication	Exact treatment procedures and data analysis steps are provided to ensure reproducibility of the study.
Randomization	Termite samples were chosen at random.
Blinding	Blinding was not possible due to a lack of expert researchers.

Reporting for specific materials, systems and methods

We require information from authors about some types of materials, experimental systems and methods used in many studies. Here, indicate whether each material, system or method listed is relevant to your study. If you are not sure if a list item applies to your research, read the appropriate section before selecting a response.

Materials & experimental systems

n/a	Involved in the study	
☒	☐	Antibodies
☒	☐	Eukaryotic cell lines
☒	☐	Palaeontology and archaeology
☐	☒	Animals and other organisms
☒	☐	Human research participants
☒	☐	Clinical data
☒	☐	Dual use research of concern

Methods

n/a	Involved in the study	
☒	☐	ChIP-seq
☒	☐	Flow cytometry
☒	☐	MRI-based neuroimaging

Animals and other organisms

Policy information about studies involving animals; ARRIVE guidelines recommended for reporting animal research

Laboratory animals	Termite species Cryptotermes secundus captured in the field: workers (unknown sex) and neotenic queens (female).
Wild animals	Cryptotermes secundus colonies were collected from dead Ceriops tagal mangrove trees near Palmerston-Channel Island (Darwin Harbor, Northern Territory, Australia; 12°30'S 131°00'E) and kept in Pinus radiata wood blocks. Shortly before extracting the tissues of interest, termite samples were killed by freezing them on ice and removing the head.
Field-collected samples	Colonies were kept in Pinus radiata wood blocks in climate rooms in Germany providing 28 °C, 70 % relative humidity and a 12 h day/night cycle. Under these conditions, colonies develop as in the field.
Ethics oversight	The Parks and Wildlife Commission, Northern Territory, the Department of the Environment, Water, Heritage and the Arts gave permission to collect (Permit number 59044) and export (PWS2016-001559) the termites. The study was conducted according to the Nagoya protocol.

Note that full information on the approval of the study protocol must also be provided in the manuscript.
